# The extent, nature, and nutritional quality of foods advertised to children in Lebanon: the first study to use the WHO nutrient profile model for the Eastern Mediterranean Region

**DOI:** 10.29219/fnr.v63.1604

**Published:** 2019-02-19

**Authors:** Lara Nasreddine, Mandy Taktouk, Massar Dabbous, Jad Melki

**Affiliations:** 1 Department of Nutrition and Food Sciences, Faculty of Agriculture and Food Sciences, American University of Beirut, Beirut, Lebanon; 2 Department of Communication Arts, Lebanese American University, Beirut, Lebanon

**Keywords:** food marketing, children, Lebanon, nutrient profiling, Eastern Mediterranean Region, media literacy

## Abstract

**Objective:**

Exposure to food marketing may influence children’s food preferences and consumption patterns and may increase the risk of childhood obesity. The WHO Office for the Eastern Mediterranean Region (EMR) has recently released a regional nutrient profile model (WHO EMR) for the purpose of regulating the marketing of food and beverages to children. This study aimed at 1) analyzing the frequency and types of food and drink advertisements during children’s viewing time in Lebanon; 2) examining the nutritional content of the advertised food products in reference to the nutrient thresholds specified by the WHO EMR model; and 3) assessing the proportion of food advertisements that included health messages.

**Design:**

This study consisted of a cross-sectional content analysis of food advertisements on local TV channels, during children’s viewing time.

**Setting:**

Three local Lebanese channels with the highest viewership among 4- to 14-year-olds were selected. Recorded broadcasts (September 2016 through January 2017) were analyzed between 3 pm and 10 pm on weekdays and between 8 am and 10 pm on weekend days.

**Results:**

Approximately 31% of advertisements were for foods or drinks. The proportion of food advertisements was the highest during children’s programs (43%) compared to general viewing (32%) and parental guidance (29%) programs. Approximately 8 out of 10 food advertisements were for products that did not meet the standards of the WHO EMR model. Of concern was the heavy advertisement of alcoholic beverages during programs for general audiences. The majority of the advertisements that comprised a health claim were for foods that did not meet the WHO EMR’s nutritional standards (79%).

**Conclusions:**

The findings of this study, which is the first to utilize the new WHO EMR profile model, should be viewed as a foundation for the development of food marketing policies aimed at reducing children’s exposure to TV food advertisements in Lebanon, a country that harbors a high burden of childhood obesity.

## Popular scientific summary

- This study demonstrated that, in Lebanon, the majority of TV food advertisements during children’s viewing time are of poor nutritional quality.
- Findings should be viewed as a foundation for the development of food marketing regulations and policies aimed at reducing children’s exposure to TV food advertisements.
- Media literacy education at early schooling stages, with emphasis on deciphering advertising messages and understanding the persuasive power of modern marketing methods, should also be promoted.

## 

Noncommunicable diseases (NCDs) represent the major cause of death in the Eastern Mediterranean Region (EMR), claiming 2.2 million lives every year, an estimate that is projected to increase to more than 3.8 million by 2030 (1–3). Evidence suggests that suboptimal diets, characterized by high intakes of fat, salt, and sugar, are among the leading risk factors for NCD mortality in countries of the region (4). Such diets are increasingly being adopted by young children and adolescents in the EMR, raising concerns about the implications of these trends on the health profile of the population in the long term (5). Studies conducted in the region suggest that children consume more total fat, saturated fat, and added sugars and fewer fruits, vegetables, and dairy products than is recommended (6–10). In addition, the prevalence of pediatric obesity has been following an alarming escalating trend, reaching epidemic proportions in many countries of the region (3, 6, 11, 12). Although several factors may affect children’s diets, one factor that has received increasing attention is food advertisement and marketing (5, 13, 14). The Institute of Medicine of the National Academies reported that food marketing influences children’s food preferences, consumption patterns, and dietary habits (15). Despite new technologies being increasingly available, TV remains the main channel for the marketing of foods to children (14), and there is strong evidence linking TV viewing and exposure to food advertisements with the consumption of energy-dense food and drinks (16, 17). However, little is known about the extent, nature, and type of food advertisements to children in countries of the EMR, although evidence suggests that TV is the most popular entertainment medium among youth in countries of the region (18, 19).

In 2010, the World Health Organization (WHO) endorsed a ‘Set of recommendations on the marketing of foods and nonalcoholic beverages to children’, advocating for 1) the collection of information on the extent, nature, and effects of food and drink marketing to children; and 2) the development of policies aimed at reducing the marketing of foods high in saturated fats, trans-fatty acids, free sugars, or salt to children (20). In 2012, the WHO Regional Committee for the EMR endorsed a regional Framework for Action for the prevention and control of NCDs, with one of its key strategic interventions aiming at ensuring ‘healthy nutrition in early life and childhood including … regulating marketing of foods and non-alcoholic beverages to children’ (21). In line with this commitment, the WHO Regional Office for the EMR developed and released, in 2017, a regional nutrient profiling model for the purpose of restricting food marketing to children (WHO EMR) (3), which was developed based on the model adopted for Europe (22). Nutrient profiling is ‘the science of classifying or ranking foods according to their nutritional composition for reasons related to preventing disease and promoting health’. It allows differentiation between foods and drinks that are more likely to be part of a healthy diet and those that are less likely to (particularly those foods that may contribute to high intakes of energy, saturated fats, trans fats, sugar, or salt) (3). The regional EMR nutrient profile model consists of a total of 18 food categories, with some subcategories (Appendix 1). When determining whether the marketing of a food product to children may or may not be ‘permitted’, the nutritional content of the food product must be cross-checked against the nutrient thresholds on a per 100 g/mL basis, as specified in the model (3). For six of the food categories, marketing to children is automatically ‘not permitted’, and hence no nutrient thresholds are specified. These categories include foods that are high in fat, sugar, and salt, such as ‘chocolate and sugar confectionaries’, ‘fruit juices’, ‘edible ices’, ‘cakes, sweet biscuits and pastries’, ‘processed meat, poultry and similar’, in addition to ‘energy drinks’ (3). The newly released nutrient profile model may be used by EMR member states as a useful tool for the assessment of the nutritional quality of foods advertised to children and the development and implementation of policies aiming at restricting the marketing of foods to this age group (20, 23, 24).

In response to the need to characterize the extent and nature of food and beverage advertisements to children in countries of the EMR, this study aims to 1) analyze the frequency and types of food and drink advertisements during children’s viewing time in Lebanon; 2) examine the nutritional content of the advertised food products in reference to the nutrient thresholds specified by the WHO EMR profile model (3); and 3) assess the proportion of food advertisements that include health messages and disclaimers. This study provides baseline data on the state of food and beverage marketing in Lebanon, and its findings will be useful for the development of regulations regarding the advertising of food products to children.

## Materials and methods

### Study design

This is a cross-sectional content analysis of food and beverage advertisements broadcasted on local TV channels during children’s viewing time in Lebanon.

Because no local Lebanese TV channels are solely dedicated to children’s programs, various local TV channels that offer general programming were selected for this study. The selection of the local TV channels was based on TV ratings and viewership share data acquired from IPSOS Lebanon, a large market research company that specializes in quantitative and qualitative marketing research, customer satisfaction research, advertising, and media (25, 26). Viewership data were available for the following age groups: 4–14 years old, 15–24 years old, 25–44 years old, and older than 45. Based on data pertinent to the 4–14 age group, channels with a viewership share exceeding 20% were included in the study. Accordingly, three out of nine local TV channels were selected for this study and will be referred to as Channel A, Channel B, and Channel C throughout the manuscript. The viewership shares of the age group 4–14 years for the selected channels were 27%, 21.1%, and 22.9%, respectively.

Recorded broadcasts from the three selected channels were purchased from IPSOS, covering the period between September 2016 and January 2017. To decrease the cost associated with purchasing and data collection, one week was randomly selected within each month, and within each week three weekdays and one weekend day were randomly selected. The month of December 2016 was excluded, given the holiday season and its potential impact on TV advertising (27).

For weekdays, the time covered all programs broadcasted between 3 pm (i.e. after the end of the school day) and 10 pm (28). For weekend days, the time covered all programs broadcasted between 8 am and 10 pm. As such, the sample covered 385 hours of TV broadcasting and included 12 weekdays and 4 weekend days.

Within these timeslots, programs that rated highest in terms of viewership among children and adolescents (4–14 years) were selected for analysis, based on IPSOS data (25). Accordingly, current affairs programs, news, cooking, and political talk shows were excluded from the analysis. The programs that were included in the analysis comprised children’s shows or C-rated programs (those specifically produced for children), G-rated (those suitable for children to view without adult supervision), and PG-rated programs (such as local soap operas or series and some entertainment programs). Of the 385 h, 226 h were analyzed for advertisements in this study (Table 1).

**Table 1:** Number of hours taped and analyzed by study week^a^ and by channel

Network channel	Week 1 (Sept 24–30, 2016)		Week 2 (Oct 24–30, 2016)		Week 3 (Nov 15–20, 2016)		Week 4 (Jan 9–14, 2017)	
	Number of taped hours	Number of analyzed hours	Number of taped hours	Number of analyzed hours	Number of taped hours	Number of analyzed hours	Number of taped hours	Number of analyzed hours
**Channel A**	35	25	35^b^	29	35	24	35	24
**Channel B**	35	18	35	14	35	17	35	16
**Channel C^c^**	0	0	35	22	35	19	35	18
**Total^d^**	70	43	105	65	105	60	105	58

a Each week included three weekdays and one weekend day. For weekdays, all programs shown between 3 pm and 10 pm were taped. For weekend days, all programs shown between 8 am and 10 pm were taped. Within the taped time slots, the programs that rated highest in terms of viewership among 4–14-year-olds were selected for analysis.

b Week 2 for Channel A was Nov 8–14, 2016, given that the recordings were not available for the week of Oct 24–30, 2016.

c Recordings were not available for Channel C during Week 1 of data collection.

d A total of 385 h were taped, of which a total of 226 h (58.7%) were analyzed.

### Data collection

Two research assistants were trained for data collection (content coding). They watched the videotapes and collected information about the advertisements using a standardized data collection sheet. They first identified the type of advertisement (food or drink vs. nonfood or nondrink advertisement). For each food or drink advertisement, the coders recorded the following information: channel, program type during which the advertisement is broadcasted, day of the week, time of day, type of food or drink being advertised, brand, whether the advertisement included health or nutrition claims, or health disclaimers (warning about excess consumption). Commercials that advertised products other than foods and drinks were not analyzed.

#### Comparison of the nutritional composition of advertised foods with the thresholds specified by the WHO EMR nutrient profile model

A list of all the advertised foods and drinks was developed. Food and beverages were then classified into 18 categories based on the WHO EMR nutrient profile model (3).

Information on the nutritional composition of each advertised food and drink was collected from their respective product labels. When the information included in the product label was not sufficient, nutrient composition information was obtained from the company’s webpage or by requesting it directly from the manufacturer. In addition, the food composition tables for the Middle East (29) and the USDA food composition database (30) were consulted, when the product label information was incomplete.

To assess whether the marketing of a food product is considered ‘permitted’ or ‘not permitted’, the nutritional content of the food product was cross-checked against the thresholds included in the nutrient profile model, namely the thresholds for energy (kcal/100 g food or drink), total fat, total sugars, added sugars, nonsugar sweeteners, saturated fat, and salt (g/100 g food or drink). As per the regional model, marketing of a food product was considered as not permitted when the food exceeded, on a per 100 g/mL basis, any of the relevant thresholds for the respective food product category (3). In addition, marketing is not permitted if the product contains >1 g/100 g of total fat in the form of industrially produced trans fatty acids or ≥0.5% of total energy in the form of alcohol.

For composite or restaurant meals that included two or more food items, all items were individually examined to assess whether the relevant nutrient criteria were met (3).

It is important to note that for 4 out of the 18 categories of the WHO EMR model, namely chocolate and sugar confectionary (Category 1), edible ices (Category 4), cakes and sweets (Category 6), processed meat and poultry (Category 14), and two subcategories (fruit juices and energy drinks) within Category 3 (beverages), marketing was considered automatically not permitted, without the need to examine the nutritional composition of the product (3).

#### Health or nutrition claims and disclaimers

In this study, health claims were considered as ‘any claim that states, suggests or implies that a relationship exists between a food category, a food or one of its constituents and health’ (31), and nutrition claims were considered as ‘any claim that states, suggests or implies that a food has particular beneficial nutritional properties due to the energy, nutrients or other substances it contains, contains in reduced or increased proportions or does not contain’ (32). The proportion of advertisements that included health or nutrition claims was calculated, by program type, as follows:

**Table:** 

(Number of advertisements with health or nutrition claims during the specific program type * 100)

(Total number of food advertisements in that type of program)

Data collection has also aimed at identifying advertisement with health disclaimers that consist of any disclosure made with the purpose of warning against the excess consumption of the food product, clarifying potential harmful consequences, or potentially misleading or deceptive statements (33).

Proportions of advertisements with disclaimers were then calculated by program type as follows:

**Table:** 

(Number of advertisements with disclaimers during the specific program type * 100)

(Total number of food advertisements in that type of program)

#### Processing and presentation of data

Intercoder reliability was assessed for the first 43 h of TV program analyzed, which, in line with other studies, represented approximately 20% of the total analyzed broadcast time (34, 35). Intercoder reliability was estimated at 97%, using the following formula (27):

**Table:** 

(Number of agreements * 100)

(Number of agreements + number of disagreements)

The collected data were entered into Microsoft Excel. The results were presented in tables or figures showing the proportions and frequency of food advertisements during C-, G-, and PG-rated programs; the percentage of food advertisements by food category; the proportions of food advertisements that do not meet the standards set by the WHO EMR nutrient profile model; and the proportions of food advertisements having health or nutrition claims or health disclaimers.

## Results

### Proportion and frequency of food advertisements

In total, 4,510 advertisements were broadcasted during the 226 h of analyzed TV programs. Of these 4,510 advertisements, 1,393 were for foods or beverages (30.9%).

**Fig. 1:**
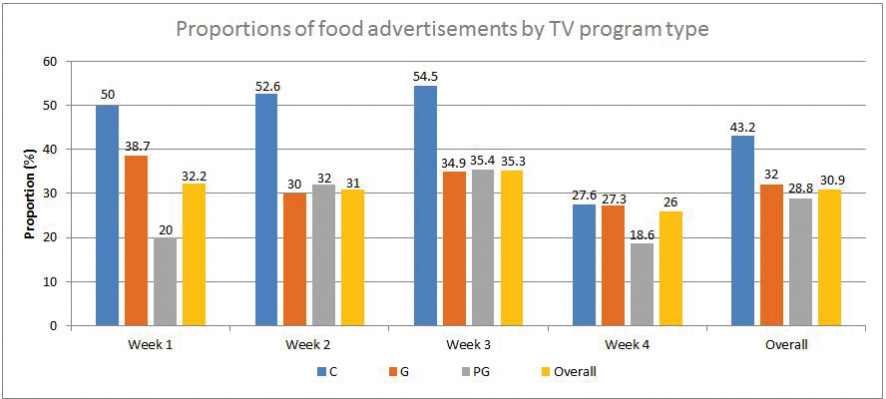
Proportions (%)^a^ of food advertisements by study week and by type of TV programs watched by children in Lebanon. ^a^Calculated as (number of food advertisements/total number of advertisements in that type of programs) × 100. C, children’s programs; G, general audience programs; PG, parental guidance programs.

The proportion of food advertisements was the highest during C-rated programs (43.2%) compared to G- and PG-rated programs (32% and 28.8%, respectively) (Fig. 1). In addition, for the three channels, the study weeks that coincided with the ‘back to school’ period in Lebanon (i.e. the months of September, October, and November) included a higher percentage of food advertisements compared to January (i.e. a regular month within the school year). This was specifically true for the children’s programs, where the proportion of food advertisements in September, October, and November (50–54.5%) was almost double that observed for January (27.6%). Overall, the average frequency of food advertisements per hour was estimated at 6.2 per hour (Table 2).

**Table 2:** Total number of advertisements, food advertisements, and hours analyzed by study week and by type of TV programs watched by children in Lebanon

	Week 1				Week 2				Week 3				Week 4				Overall			
	Total ads	Food ads	Hours	Freq/hour	Total ads	Food ads	Hours	Freq/hour	Total ads	Food ads	Hours	Freq/hour	Total ads	Food ads	Hours	Freq/hour	Total ads	Food ads	Hours	Freq/hour
**C**	22	11	2.93	3.8	19	10	2.07	4.8	11	6	3.08	1.9	29	8	2.90	2.8	81	35	10.98	3.2
**G**	442	171	24.87	6.9	808	242	37.45	6.5	651	227	29.64	7.7	652	178	41.22	4.3	2553	818	133.18	6.1
**PG**	270	54	15.10	3.6	582	186	24.57	7.6	653	231	26.73	8.6	371	69	15.90	4.3	1876	540	82.30	6.6
**Overall**	734	236	42.90	5.5	1409	438	64.09	6.8	1315	464	59.45	7.8	1052	255	60.02	4.2	4510	1393	226.46	6.2

*Note*: freq, frequency; C, children’s programs; G, general audience programs; PG, parental guidance programs.

### Type of food advertisements

Out of the 18 categories included in the EMR nutrient profiling model, 12 were advertised during the analyzed TV programs (Table 3). The six categories that were not advertised included edible ices; yoghurts, sour milk, cream, and similar foods; bread, bread products, and crispbreads; processed fish; fresh and frozen fruit, vegetables, and legumes; and processed fruit, vegetables, and legumes. Furthermore, apart from the 18 food categories included in the EMR model, two additional food categories were advertised during the analyzed TV programs, namely alcoholic drinks and coffee (Table 3).

**Table 3:** Percentage of food advertisements^a^ by food category and by type of TV programs

Category	Food category	C *n* (%)	G *n* (%)	PG *n* (%)	Overall *n* (%)
**Food categories based on the WHO EMR model**					
1	Chocolate and sugar confectionery, energy bars, and sweet toppings and deserts	12 (33.3)	140 (16.6)	56 (10.1)	208 (14.5)
2	Savory snacks	6 (16.7)	56 (6.6)	42 (7.6)	104 (7.2)
3	Beverages				
a	Fruit juices	-	7 (0.8)	4 (0.7)	11 (0.8)
b	Vegetable juices	-	-	-	-
c	Milk drinks	5 (13.9)	28 (3.3)	19 (3.4)	52 (3.6)
d	Energy drinks	-	31 (3.7)	11 (2.0)	42 (2.9)
e	Other beverages	4 (11.1)	37 (4.4)	18 (3.2)	59 (4.1)
4	Edible ices (ice cream, iced lollies, sorbets, and frozen yogurt)	-	-	-	-
5	Breakfast cereals	-	1 (0.1)	3 (0.5)	4 (0.3)
6	Cakes, sweet biscuits, and pastries; other sweet baked goods, and dried mixes for making such goods	-	142 (16.8)	113 (20.4)	255 (17.8)
7	Yoghurts, sour milk, cream, and other similar foods	-	-	-	-
8	Ready-made and convenience foods and composite dishes^b^	-	11 (1.3)	17 (3.1)	28 (1.9)
9	Cheeses	9 (25.0)	43 (5.1)	19 (3.4)	71 (4.9)
10	Butter and other fats and oils	-	19 (2.2)	-	19 (1.3)
11	Bread, bread products, and crispbreads	-	-	-	-
12	Fresh, dried, or cooked pasta, rice, and grains	-	2 (0.2)	-	2 (0.1)
13	Fresh and frozen meat, poultry, fish, and similar	-	57 (6.7)	74 (13.3)	131 (9.1)
14	Processed meat, poultry, and similar	-	18 (2.1)	22 (4.0)	40 (2.8)
15	Processed fish	-	-	-	-
16	Fresh and frozen fruit, vegetables, and legumes	-	-	-	-
17	Processed fruit, vegetables, and legumes	-	-	-	-
18	Sauces and dressings	-	31 (3.7)	14 (2.5)	45 (3.1)
**Additional food categories**					
19	Alcoholic drinks	-	134 (15.9)	94 (16.9)	228 (15.9)
20	Coffee	-	88 (10.4)	49 (8.8)	137 (9.5)

*Note*: C, children’s programs; G, general audience programs; PG, parental guidance programs; EMR, Eastern Mediterranean Region.

a Calculated as (number of food advertisements/total number of advertisements in that type of programs) × 100.

b Category 8 includes ready-made and convenience foods and composite dishes (pizzas, pastas, lasagna, ready meals including traditional composite dishes, ready-made sandwiches, canned or packaged soups and stews, rice dishes, mixes and dough, falafel, and hummus).

The most-advertised food category during C-rated programs was chocolate and sugar confectionary (33.3% of food advertisements), followed by cheeses (25%), savory snacks (16.7%), milk drinks (13.9%), and ‘other beverages’ (11.1%), which included soft drinks and mineral water (Table 3).

During the G-rated programs, chocolate and sugar confectionary together with cakes, biscuits and pastries represented 33.4% of food advertisements, followed by alcoholic drinks (15.9%) and coffee (10.4%). Similarly, during the PG-rated programs, chocolate and sugar confectionary together with cakes, biscuits, and pastries represented 30.5% of food advertisements, followed by alcoholic drinks (16.9%) and fresh and frozen meats (13.3%).

### Comparison of the nutritional composition of the advertised food products with the thresholds specified by the EMR nutrient profile model

Of the 1,393 food advertisements broadcasted during the study period, 365 commercials (26.2%) were for alcoholic drinks or coffee, while 1,028 (73.8%) were for foods and non-alcoholic beverages. These 1,028 advertisements were for 68 specific food products. For 91.2% of these products, the nutrient composition information was obtained from the product label or website, while for the remaining 8.8%, the information was obtained from food composition databases (29, 30).

When referring to the 18 categories of the WHO EMR model and when cross-checking the nutrient composition of the advertised foods against the model’s thresholds, only 16.3% of the advertisements were assessed as ‘permitted’ for marketing to children (Table 4). These permitted advertisements were for the following types of products: dried pasta, vegetable oils, mineral water, fresh poultry, and tomato paste (no salt). The remaining 83.7% of the advertisements were for food products that exceeded one or more of the relevant thresholds specified by the EMR nutrient profiling model, and thus their marketing to children ought not to be permitted.

**Table 4:** Proportion^a^ of TV food advertisements assessed as ‘permitted’ or ‘not permitted’ for marketing to children, based on the WHO EMR model

	Number of food ads	Ads permitted *n* (%)	Ads not permitted *n* (%)
**Based on the 18 food categories included in the WHO EMR model**			
**C programs**	35	0 (0)	35 (100.0)
**G programs**	596	88 (14.8)	508 (85.2)
**PG programs**	397	80 (20.2)	317 (79.8)
**Overall**	1,028	168 (16.3)	860 (83.7)
**Based on all the advertised food categories, including alcohol and coffee**			
**C programs**	35	0 (0.0)	35 (100.0)
**G programs**	818	88 (10.8)	730 (89.2)
**PG programs**	540	80 (14.8)	460 (85.2)
**Overall**	1,393	168 (12.1)	1,225 (87.9)

*Note*: C, children’s programs; G, general audience programs; PG, parental guidance programs; EMR, Eastern Mediterranean Region.

a Calculated as (number of food advertisements/total number of advertisements in that type of programs) × 100.

When examining the proportion of permitted food advertisements by program type, Table 4 shows that 100% of the advertisements shown during C-rated programs were for foods that did not meet the standards set by the WHO EMR model. For the G-rated and PG-rated programs, 85.2% and 79.8% of the food advertisements were for foods that did not meet the standards specified by the profile model.

### Health and nutrition claims and disclaimers

Table 5 shows the proportion of food advertisements that included a health or nutrition claim or a health disclaimer. Overall, 17.9% of the food advertisements included health or nutrition claims, varying between 14.3% and 19.2% for the C- and G-rated programs, respectively.

**Table 5:** Proportions^a^ (%) of food advertisements with health and nutrition claims or health disclaimers, by type of TV program

	Health and nutrition claims *n* (%)	Disclaimers *n* (%)
**C programs**	5 (14.29)	1 (2.86)
**G programs**	157 (19.19)	31 (3.79)
**PG programs**	87 (16.11)	22 (4.07)
**Overall**	249 (17.88)	54 (3.88)

*Note*: C, children’s programs; G, general audience programs; PG, parental guidance programs.

a Calculated as (number of food advertisements/total number of advertisements in that type of programs) × 100.

During C-rated programs, the health and nutrition claims focused on healthy growth or the provision of vitamins and minerals. Interestingly, during C-rated programs, all of the advertisements that comprised a health or nutrition claim were for foods that did not meet the nutritional standards set by the EMR nutrient profile model (data not shown).

During the G-rated programs, the most frequent health or nutrition claims shown in food advertisements were for low content of calories, fat, or sugar; healthy growth in children; and the promotion of weight loss. Of the advertisements that contained a health or nutrition claim, only 20.7% were for food items that met the WHO EMR nutrient profile standards (data not shown).

During the PG-rated programs, the most frequent health or nutrition claims shown in food advertisements were for low content of calories, fat, or sugar and for healthy growth. Of the advertisements that contained a health or nutrition claim, only 15% were for food items that met the WHO EMR nutrient profile standards (data not shown).

Health disclaimers were included in only 2.9% of the advertisements shown during C-rated programs and in 3.8 and 4.1% of those shown during the G- and PG-rated programs, respectively. During C-rated programs, the disclaimers targeted the importance of brushing teeth after the consumption of sweet products. For the G- and PG-rated programs, the disclaimers cautioned against mixing alcohol with energy drinks and recommended drinking responsibly (data not shown).

## Discussion

To our knowledge, this study is among the first in the region to comprehensively analyze the extent and types of food advertisements broadcasted on TV during children’s viewing time. It is also the first to utilize the newly released WHO EMR nutrient profile model in assessing the nutritive quality of food and beverages advertised to children.

The study results showed that approximately 31% of TV advertisements were devoted to foods and drinks in Lebanon, a country that does not have current regulations or restrictions on the marketing of foods and beverages to children. The observed proportion of food advertisements (31%) is higher than what was observed in Spain, New-Zealand, the UK, and Norway (14.6–25.5%) (28, 36–39), where regulations on the marketing of food to children are enforced (40–43), while being similar to Iran, Turkey, and Australia (31–32%) (27, 44, 45), which differ in their degree of enforcement of food advertisement regulations (46–49). The study results have also shown that the highest proportion of food advertisements was observed during C-rated programs, compared to the G- and PG-rated programs (43.2 vs. 28.8–32%, respectively). It is however important to note that, in our study, only 11 h of the total 226 analyzed hours were devoted to C-rated programs, highlighting the scarcity of such programs on Lebanese TV channels. Thus, in our study, the majority of food-related advertisements came from G-rated programs, with a total of 818 advertisements, at a frequency of six per hour. Few other studies have examined the extent of food advertisements by type of TV program (27, 36). Our findings are similar to what has been reported by Zuppa et al. (2003) in Australia, where general viewing programs were considered the highest contributor to children’s exposure to food commercials (27). A study conducted in Spain also reported a high contribution of general interest TV programs to food advertisement exposure among children (36).

In line with other studies (44, 50), our findings showed that chocolates and sugar confectionaries, as well as cakes and sweets, were among the most frequently advertised food categories during the three types of TV programs. Savory snacks, cheeses, and sweetened beverages were also among the commonly advertised food categories. Even though other studies have shown that fast food and breakfast cereals were frequently marketed to children (13, 27, 37, 51–54), this was not the case in our study. This is largely expected, as cereals are not typical breakfast items in Lebanon (55). In agreement with previous studies, the advertising of healthier options of foods such as fruits, vegetables, and legumes was completely absent (27, 37, 45, 56). These results suggest that television food advertising during children’s viewing times is disproportionately promoting the consumption of foods high in fat, sugar, and salt, thus promoting unhealthy dietary patterns in children (27).

Based on the WHO EMR nutrient profile model, our results showed that approximately 8 out of 10 food advertisements were for foods or drinks that did not meet the nutrition standards set by the model. These findings are in line with those reported by Batada et al. (13) in the United States, where 9 out of 10 food advertisements broadcasted during Saturday children’s television programs were for foods of poor nutritional quality, based on nutrition standards derived from the National Alliance for Nutrition and Activity’s Model Local School Wellness Policies (57–59). Lower proportions were reported from Spain, the UK, Canada, and New Zealand, where 54.5–66.3% of food advertisements broadcasted during children’s TV airtime were for foods considered ‘less healthy’ (28, 37, 60). Our findings were in stark contrast with what has been recently reported from Norway, where only 2 out of 10 food advertisements shown to children were for foods that were high in fat, sugar, or salt. This may be explained by the strict and effective food marketing regulations in Norway, which were implemented in 1990 and further strengthened in 2014 to restrict the marketing of foods to children under the age of 13 (39, 40, 61).

It is worth noting that compared to the proportion of advertisements with health or nutrition claims (17.9%), very few commercials included heath disclaimers (3.9%). Interestingly, all the advertisements that comprised a health or nutrition claim and that were broadcasted during the C-rated programs were for foods that did not meet the nutritional standards of the WHO EMR model. This was also true for the majority of the commercials shown during the G- and PG-rated programs. In line with other studies, these findings suggest that the food advertisements broadcasted during children’s viewing times in Lebanon may be providing misleading nutrition information to children and their caregivers (62–65).

A particularly concerning finding in this study was the frequent advertisement of alcoholic drinks during and around the G- and PG-rated programs. It may be contested that adult supervision and interference during PG-rated programs may dampen the impact of advertisements on children’s assimilation and behavior (66, 67). However, the G-rated programs, which may be watched by children without adult supervision, included the same high proportion of alcohol advertisements as the PG-rated programs. This is alarming, given that several studies have shown that exposure to alcohol-related advertising may increase and encourage drinking behavior among youth (68, 69). A systematic review of longitudinal studies showed that exposure to commercial advertisement of alcohol was consistently associated with the likelihood that adolescents would start to drink alcohol and to drink more if they were already using alcohol (68). Adolescent drinking may lead to other high-risk behaviors such as smoking and drug use (70), while also being linked with sleeping disorders, social misbehavior, and mental health problems (71, 72). As for coffee, which was also found to be heavily advertised during children’s viewing time in Lebanon, a previous study has shown that its consumption among children has been positively linked with stress, anxiety, and depression (73).

Overall, the results of this study suggest that children in Lebanon are heavily exposed to advertisements that market unhealthy dietary choices and practices on TV. Previous studies have shown that the amount of time spent watching TV and the exposure to food commercials may increase the child’s request for, purchase, and consumption of the foods and drinks advertised on TV (15, 53, 74), as well as the development of unhealthy eating habits, characterized by higher intakes of sugar, fat, salt, and alcohol (75). The adoption of such eating habits early in life may increase the risk of obesity and cardiometabolic abnormalities such as dyslipidemia, hyperglycemia, and elevated blood pressure, thus increasing the risk for adult-onset NCDs (76). Available studies in Lebanon have shown that the diet of children is increasingly shifting from the traditional Lebanese dietary pattern – characterized by high intakes of fruits, vegetables, pulses, grains, and olive oil (77) – towards a westernized dietary pattern that is characterized by high consumption of red meat, fast food, sweets, and sugar-sweetened beverages (77). Of more concern is the alarming increase in childhood obesity, which was found to almost double in the past decade (78), reaching 13.2% among 6–19-year-olds in Lebanon (6), and the high prevalence of metabolic syndrome in obese Lebanese children and adolescents (21.2%) (79–81). Together, these findings highlight the need for public health interventions aimed at promoting healthier dietary options among youth. Changes in the television advertising environment are acknowledged as one of the most cost-effective ways for encouraging healthier food choices in this age group (20, 27, 82, 83). In this context, in 2007, the UK government was successful in banning the advertising of less healthy products in programs with a large audience of children, a measure that was expanded in 2009 to include all food advertising on children’s channels (28, 84). This has reduced children’s exposure to advertisements for less healthy products in the UK by over one-third in 2 years (28, 84) and has decreased per capita expenditure on sweetened beverages and high fat, sugar, and salt foods (85).

The findings of this study should be considered in light of the following limitations. First, this study may underestimate children’s exposure to food advertisements because television is only one of many different media through which advertisers are able, nowadays, to market food products to this age group. However, despite new technologies, TV remains the main channel for the marketing of food and drinks to children (14). In Lebanon, television constitutes the dominant advertising platform, with television advertising accounting for almost 38% of total advertising spending in 2013 (86). Second, in this study, we have only selected local TV channels for the analysis of food commercials, even though satellite viewing may be another medium for exposure to such advertisements. However, household audience views have shown that local channels, which broadcast culture-specific programs, are the most viewed in Lebanon (86). Third, it is important to note that the results of this study are snapshots of TV food advertising during the period under study and hence may not be representative of yearlong food marketing in Lebanon. In this context, we made sure to sample different months, different weeks, and different days to increase the representativeness of the collected data, but longer studies capturing different seasons are needed to provide a more complete picture of food advertising in Lebanon.

## Conclusion

This study has mapped food advertisements broadcasted during television programs of particular appeal to children and adolescents in Lebanon and showed that approximately 8 out of 10 commercials are for foods that do not meet the nutritional standards set by the WHO EMR. As this may have implications on dietary habits, weight gain, and disease risk later in life, the results of this study should be viewed as a foundation for the development of food marketing policies aimed at reducing children’s exposure to TV food advertisements in Lebanon. The WHO EMR profiling model may be used by policy makers as a tool to limit the marketing of foods that are high in fat, sugar, and salt during children’s viewing times. The results of this study have also documented worrisome findings pertinent to the marketing of alcohol during children’s viewing times, thus highlighting the need for policies and legislative interventions to regulate alcohol advertising on TV. In a country that is currently witnessing a nutrition transition and where the traditional diet is progressively eroding, particularly among youth, television may be used as an educational medium that promotes and encourages the consumption of healthier food options, including those that are part of the Lebanese traditional diet. In addition to developing food marketing policies to protect children, this study provides a strong argument for promoting media literacy education at the early stages of schooling, with special emphasis on deciphering advertising messages and understanding the persuasive power of modern marketing methods. Media literacy is a mode of education that promotes critical thinking and awareness of the power of media in society. It helps individuals better protect themselves from misleading and often harmful marketing messages. Today, most developed countries, especially within the European Union, have national media literacy policies for schools and universities. Unfortunately, Lebanon and most Arab countries still lag behind on this front, despite significant advances at the university level prompted by the Media and Digital Literacy Academy of Beirut and the UNESCO/United Nations Alliance of Civilizations (87, 88).

## Appendix

**Appendix 1:** WHO EMRO nutrient profile model

Food category	Marketing not permitted if exceeds, per 100 g^a^						
	Total fat (g)	Total sugars (g)	Added sugars (g)	Nonsugar sweeteners (g)	Energy (kcal)	Saturated fat (g)	Salt (g)
1. Chocolate and sugar confectionery, energy bars, and sweet toppings and desserts	Not permitted						
2. Savory snacks			0				0.1^b^
3. Beverages							
a) Fruit juices	Not permitted^c^						
b) Vegetable juices			0				0.1
c) Milk drinks^d^	2.5		0	0			
d) Energy drinks^e^	Not permitted						
e) Other beverages			0	0			
4. Edible ices	Not permitted						
5. Breakfast cereals^f^	10	15					1.6
6. Cakes, sweet biscuits, and pastries; other sweet baked goods and dried mixes for making such goods	Not permitted						
7. Yoghurts, sour milk, cream, and other similar foods	2.5	10				2	0.1
8. Ready-made and convenience foods and composite dishes	10	10			225	4	1
9. Cheese	20						1.3
10. Butter and other fats and oils						20	1.3
11. Bread, bread products, and crispbreads^f^	10	10					1
12. Fresh, dried, or cooked pasta, rice, and grains	10	10					1
13. Fresh and frozen meat, poultry, fish, and similar							0.1
14. Processed meat, poultry, and similar	Not permitted						
15. Processed fish						2	1.7
16. Fresh and frozen fruit, vegetables, and legumes	Permitted						
17. Processed fruit, vegetables, and legumes	5	10	0				1
18. Sauces and dressings	10		0				1

Adapted from the WHO EMRO nutrient profile model (3).

Marketing is not permitted if the product contains >1 g per 100 g total fat in the form of industrially produced trans fatty acids or ≥0.5% of total energy in the form of alcohol.

a The food products should, where possible, be assessed as sold or as reconstituted (if necessary) according to the manufacturer’s instructions.

b Salt equivalent.

c This is in line with the WHO guidelines on sugar intake for children and adults (89), as fruit juices are a significant source of free sugars for children. However, it is recognized that countries, according to national context and national food-based dietary guidelines, may take the decision to permit the marketing of 100% fruit juices in small portions.

d Follow-up formulas and growing-up milks are not covered by this model. It should be noted that World Health Assembly Resolution WHA39.28, adopted in 1986, states that the practice of providing infants with specially formulated milks (so-called follow-up milks) is not necessary. Further, any food or drink given before complementary feeding is nutritionally required may interfere with the initiation or maintenance of breastfeeding and should, therefore, be neither promoted nor encouraged for use by infants during this period.

e There is no agreement on a definition of energy drinks. However, such a category of drinks includes a variety of nonalcoholic beverages. While caffeine is considered the main ingredient, a number of other substances are often present. The most common of these include guarana, taurine, glucuronolactone, and vitamins. A common feature is that these beverages are marketed for their actual or perceived effects as stimulants, energizers, and performance enhancers.

f For this category, countries may choose to include a threshold for minimum dietary fiber content, for example ≥6 g dietary fiber.

*Note*: EMRO, Eastern Mediterranean Regional Office.
